# Heavy Metals Exposure and Hygienic Behaviors of Workers in Sanitary Landfill Areas in Southern Thailand

**DOI:** 10.1155/2016/9269210

**Published:** 2016-05-23

**Authors:** Somsiri Decharat

**Affiliations:** Department of Industrial Hygiene and Health Science, Faculty of Health and Sports Science, Thaksin University, Phatthalung 93210, Thailand

## Abstract

*Objectives*. The main objective of this study was to assess the cadmium and lead exposure levels in subject workers that work in sanitary landfill areas in southern Thailand. The study evaluated the blood cadmium and lead levels in terms of their possible role in worker contamination and transfer of cadmium and lead to the body.* Materials and Methods*. A cross-sectional study was conducted with 114 subjects. Whole blood samples were collected to determine cadmium and lead levels by graphite furnaces atomic absorption spectrometer chromium analyzer.* Results and Discussion*. The mean blood cadmium levels and blood lead levels of subjects workers were 2.95 ± 0.58 *μ*g/L (range 1.58–7.03 *μ*g/L) and 8.58 ± 2.58 *μ*g/dL (range 1.98–11.12 *μ*g/dL), respectively. Gender, income, smoked cigarettes, work position, duration of work, personal protective equipment (PPE), and personal hygiene were significantly associated with blood cadmium level and blood lead levels (*p* < 0.001 and *p* < 0.001). A multiple regression model was constructed. Significant predictors of blood cadmium levels and blood lead levels included smoked cigarettes, hours worked per day, days worked per week, duration of work (years), work position, use of PPE (mask and gloves), and personal hygiene behavior (ate snacks or drank water at work and washed hands before lunch).* Conclusion*. The elevated body burden of toxic metals in the solid waste exposure of subject workers is an indication of occupational metal toxicity associated with personal hygiene practices.

## 1. Introduction

A total of 127 waste disposal sites in Thailand include landfills (88.19%), incinerator (2.63%), and integrated systems (9.45%), respectively. 85% of all landfills are presently operated and 50% of all located in the central part of Thailand. The most of waste disposal sites are disposed legally in landfills. Landfill is the end of disposal site to waste, intended to minimize the impact of solid waste on human health and environment [1]. Leaching of harmful substances to the vicinity is protected by the use of liner systems [2]. Landfill classification is influenced by region, nation, site area, population characteristics, type of waste, and amount of waste. According to [1], landfills can be classified into six major types including (1) semicontrolled dump, (2) controlled dump, (3) engineered landfill, (4) sanitary landfill, (5) sanitary landfill with top seal, and (6) controlled contaminant release landfill. Apart from that, transferred station is another type of landfills currently used in Bangkok [3]. In many countries, scatter pickers (scavengers) support themselves through sorting and recycling of secondary materials at such landfills. These scavengers may have a high health risk from contact with human fecal matter, exposure to toxic substances, and contact with pathogenic organisms (e.g., needles and bandages).

In addition, exhaust fumes of waste collection trucks traveling to and from disposal sites, dust from disposal operations, and open burning of waste all contribute to occupational health problems [4, 5]. Blood cadmium levels were significantly associated with the paper sorters and solid waste handlers and blood mercury levels only among paper sorters. While elevated, all levels measured were within acceptable ranges for long-term health [6, 7]. The workers were exposed to air lead concentrations as high as 2500 *μ*g/m^3^, compared to the standard of 1 *μ*g/m^3^. The incinerator workers were exposed to air lead levels as high as 2500 *μ*g/m^3^, compared to the standard of 1 *μ*g/m^3^. The incinerator workers who worked in incinerator plant had blood lead level 11.00 *μ*g/dL, compared to the heating plant control group of 7.4 *μ*g/dL.

Workers who worked in cleaning the precipitates had higher blood lead levels (16 *μ*g/dL) and the workers who did not regularly wear their protective device had highest levels (28.7 *μ*g/dL). Blood lead levels of workers who wore respiratory protection during cleaning of the precipitates were significantly lower, when compared to those who did not. While the blood levels of incinerator workers were higher than for heating plant workers, the levels for both groups were below the USA OSHA health safety action limit considered (in 1989) to be unacceptable in the workplace of 40 *μ*g/dL [8].

The objective of this study was to determine and evaluate the level of blood cadmium and blood lead among subject workers exposed to cadmium and lead from behaviors in terms of their possible role in worker contamination and transfer of cadmium and lead to the body.

## 2. Materials and Methods

The study was designed as a cross-sectional study. Whole blood samples were collected from workers in five sanitary landfill areas in southern Thailand, between August and October 2014. This research was approved by the Ethics Committee of the Institute of Research and Development, Thaksin University.

### 2.1. Sample Collection

The population in this study included the subject workers (30 office workers, 30 drivers, and 54 garbage workers, resp.) who work at sanitary landfill sites in southern Thailand. The landfill sites are located at 2 sites in Nakhon Si Thammarat province, 2 sites in Songkha province, and 1 site in Phatthalung province. Nakhon Si Thammarat province, Songkha province, and Phatthalung province are located in middle southern Thailand that presented the landfill area supporting the amount of solid waste in municipalities increasing every year (more than 1000 tons a day in 2015), and all of areas have an approximate area of more than 24,000 square kilometers. All of landfill was managed by Local Administrative Organization. The sampling was selected by purposive selection. All of subjects were formal workers. The formal workers who worked at 5 sanitary landfill areas were 456 persons to determine the sample size by using the 25 percent of the population. 100 percent of the subject workers (114 persons) agreed to participate in this study. The inclusion criteria for the subject workers were formal workers and of age 20–60 years, in occupational contact for at least one year. In this study, classification of risk exposure is in two groups including (1) direct group including the subject workers who work in the process of mixing domestic, transferring waste, sorting, and dumping at landfill; (2) indirect group including drivers and office workers. Questionnaire was used by interviewing in subject workers group. Face-to-face interview and walkthrough survey were used by collecting general information on the subject workers. From each sample, one aliquot (4 mL) was kept in an acid cleaned plastic test tube and was analyzed for cadmium and lead content with the GFASS [9].

### 2.2. Questionnaire

In the questionnaire, information on the following variables was collected: general information, work characteristics (e.g., office workers, drivers, and garbage workers), and personal hygiene, as a result of managing sanitary landfill processes, which lasted three months. The patterns of behavior reported in the questionnaire were confirmed by directing observation. Whole blood was collected from the study participants at the end of their work shift.

### 2.3. Sample Preparation

#### 2.3.1. Whole Blood Cadmium Preparation

Blood cadmium sample was transferred into 100 mL conical flash. The EDTA bottle was rinsed with little HNO_3_ and transferred into the 100 mL conical flash. Then, two mL perchloric acid and six mL nitric acid (1 : 3). The conical flash was covered with an evaporating dish. Next, the samples were mixtures digested at low temperature. The digest was made up to 20 mL with deionized water in a 20 mL standard flash. The measurement was made at 288.8 nm. Under the operation conditions were previously described in detail [9].

#### 2.3.2. Calibration, Recovery, and Reproducibility of Whole Blood Cadmium Determination

Graphite furnace atomic absorption spectrophotometer (GFASS), Hitachi Model Z-8200 (Hitachi Ltd., Tokyo, Japan), was used for measuring the blood cadmium levels and lead levels. Field water blank samples were frozen and shipped on dry ice in all of the analyses as a quality control. These field blanks of cadmium were analyzed by using the same method. Standard cadmium solution of 100 mg/L was prepared by dilution with 0.2% nitric acid from a stock solution of 1000 mg/L (Merch, FRG). A series standard of blood cadmium level was prepared at 2.5, 5.0, and 10.0 *μ*g/L of Cd. The correlation coefficient (*r*) between the cadmium concentration in the authentic cadmium solution and the absorption intensity was 0.9996. The method limit of determination (LOD) of blood cadmium levels analyzed by GFASS was 0.5 *μ*g/L.

#### 2.3.3. Whole Blood Lead Preparation

Blood lead sample preparation place 2 mL of whole blood into a culture tube. Start a reagent blank at this point with 2 mL deionized water and 0.8 mL of diluted solution (ammonium pyrrolidine dithiocarbamate) was added. The samples were mixed on a rotary vibration mixer for 10 seconds. Then, 2 mL of water saturated with methyl isobutyl ketone (MIBK) was added to the mixture. Each culture tube was capped and rotated on a rotary vibration mixer for 2 minutes. Next, the samples were centrifuged at 2,000 rpm for 10 minutes. Next, the samples were analyzed with lead ammonium pyrrolidine dithiocarbamate (Pb-APDC) solution in MIBK within 2 hours of extraction [10]. The absorbance was measured at a wavelength of 228.8 nm.

#### 2.3.4. Calibration, Recovery, and Reproducibility of Whole Blood Lead Determination

Whole blood lead determination was calibrated by preparing a series of standard additions, that is, 0, 5, 10, 20, 30, and 50 *μ*g/dL. The correlation coefficient (*r*) between the lead concentration in the authentic lead solution and the absorption intensity was 0.9995. The limit of quantitation was defined as the lower limit for precise quantitative measurement, given as the value of the signal blank plus 10 times the SD of the blank (3SD_b_/S) at 4 *μ*g/dL. A known sample containing added Seronorm*™* Trace Elements (SGAB AS-Whole Blood L-2, Sero, Germany), 0.5, 1.0, and 2.0 *μ*g/dL, was used in determining within-day accuracy and between-day precision of the method. The RSDs (100 × SD/mean) were calculated for 10 days for between-day precision. The accuracy of the overall method is about 84.70.5%, and the calculated precision was within 10% RSD.

### 2.4. Statistical Analysis

Descriptive statistics were used to present the blood cadmium levels and blood lead levels test results. The independent *t*-test was used to compare the means of continuous variables. Normally distributed data group means were compared using Student's *t*-test or ANOVA for 2 or more than 2 groups, respectively. Multiple linear regression analysis was used to evaluate the effects on blood cadmium levels and blood lead levels of the general characteristics, work characteristics, occupational lifestyle, personal protective equipment (PPE), and personal hygiene of workers. Used PPE and personal hygienic practice were treated as dummy variables (yes/no and always/sometimes) in the model. A *p* value < 0.05 was considered statistically significant.

## 3. Results

### 3.1. General Characteristics of the Subjects

One hundred and fourteen subjects participated in the present study. Most subjects (74.6%) were male and most subjects (57.9%) were aged between 35 and 44 years. Most subjects had secondary level and the mean income was 7,569 baht per month. Most subjects smoked cigarettes (56.1%) and drank alcoholic beverages (57.9%) (Table 1).

### 3.2. Work Characteristics

Most subjects (85.1%) had been working for ≥10 years. Most subjects worked for >8 hours per day and 6 days per week, at 60.5 and 71.9%, respectively. Most subjects (47.4%) sorted general waste (Table 2).

### 3.3. Occupational Lifestyles, PPE, and Personal Hygiene of Workers

The majority (85.0%) started working at age < 15 years. 26.3% were drivers, 47.3% were garbage workers, and 26.3% worked in an office. Subject workers (25.4%) did not use a cotton mask to protect themselves from air pollutants, 64.0% of workers used gloves when collecting or handling waste, and 60.5% of workers used aprons when collecting waste.

Forty-six point four percent always ate snacks or drank water while working. Over half of the workers (58.8%) always washed their hands before lunch. Workers (20.5%) sometimes changed their clothes after worked. Before going home, none of the workers took a shower or regularly changed their clothes (Table 3). The blood cadmium levels and blood lead levels of the subject workers are shown in Table 4.

It was found that the mean blood lead levels and blood cadmium levels and age, income, smoke cigarettes, duration of work, hours worked per day, days worked per week, work position, use of PPE (mask, gloves, and apron), eating snacks or drinking water during work, and washing hands before lunch were significantly different, at *p* < 0.005.

Workers who had worked ≥10 years had significantly higher blood cadmium levels and blood lead levels than those who had worked <10 years (*p* < 0.001 and *p* < 0.001, resp.). Workers who had worked >6 days per week had significantly higher blood lead levels than those who had worked 6 days per week (*p* < 0.001).

The results indicated that the mean blood cadmium levels and blood lead levels among work positions were significantly different (*p* < 0.001). Workers who used cotton mask and/or gloves had significantly lower blood cadmium levels and blood lead levels than those who did not. Workers who always ate snacks had significantly higher blood cadmium levels and blood lead levels than those who sometimes ate them. Workers who always washed their hands after work had significantly lower blood cadmium levels and blood lead levels than those who sometimes did so (Table 5).

To predict the blood cadmium levels and blood lead levels of workers, a multiple regression model was constructed, as shown in Table 6. To predict the blood cadmium levels and blood lead levels of subject workers, a regression model was presented, as in equation 1. There were significant influences between the independent variables and the blood cadmium levels; the entire *R* ^2^ was 0.418, indicating that the blood cadmium levels could be interpreted into 41.8% of the independent variables. Smoking, days worked per week, position, and using mask affected the blood cadmium levels (*p* < 0.001, *p* < 0.001, *p* < 0.001, and *p* < 0.001, resp.). For blood lead levels, the entire *R* ^2^ was 0.450, indicating that the blood lead levels could be interpreted into 45.0% of the independent variables. Smoking, position, and using mask affected the blood lead levels (*p* < 0.001, *p* < 0.001, and *p* < 0.001, resp.).

In equation 2, the entire *R* ^2^ was 0.458, indicating that the blood cadmium levels could be interpreted into 45.8% of the independent variables. Days worked per week and using gloves affected the blood cadmium levels (*p* < 0.001 and *p* = 0.031, resp.). For blood lead levels, the entire *R* ^2^ was 0.378, indicating that the blood lead levels could be interpreted into 37.8% of the independent variables. Position and using gloves affected the blood lead levels (*p* < 0.001 and *p* = 0.011, resp.).

In equation 3, the entire *R* ^2^ was 0.401, indicating that the blood cadmium levels could be interpreted into 40.1% of the independent variables. Smoking and eating snacks or drinking water at work affected the blood cadmium levels (*p* < 0.001 and *p* < 0.001, resp.). For blood lead levels, the entire *R* ^2^ was 0.530, indicating that the blood lead levels could be interpreted into 53.0% of the independent variables. Days worked per week, duration of work, and eating snacks or drinking water at work affected the blood lead levels (*p* < 0.001, *p* < 0.001, and *p* = 0.021, resp.).

In equation 4, the entire *R* ^2^ was 0.569, indicating that the blood cadmium levels could be interpreted into 56.9% of the independent variables. Smoking, days worked per week, position, and washed hands before lunch affected the blood cadmium levels (*p* < 0.001, *p* < 0.001, *p* < 0.001, and *p* < 0.001, resp.). For blood lead levels, the entire *R* ^2^ was 0.391, indicating that the blood lead levels could be interpreted into 39.1% of the independent variables. Days worked per week, duration of work, and position affected the blood lead levels (*p* < 0.001, *p* < 0.001, and *p* < 0.001, resp.).

## 4. Discussion

### 4.1. Heavy Metals Levels

Blood cadmium level is an indicator of recent exposure although, upon long-term exposure (i.e., decades). The most commonly, blood levels was used maker for lead exposure and reflects a combination of exposure during the last month and several years back in time [11]. Most subject workers had cadmium levels <5 *μ*g/L [12], the ACGIH-recommended biological exposure index for cadmium in blood.

Alfvén et al. [13] reported that the group with the highest blood cadmium levels (≥1 *μ*g/L) had a 4-fold greater risk of having renal dysfunction compared with the group with the lowest blood cadmium levels (≤0.5 *μ*g/L).

All subject workers had blood lead levels <30 *μ*g/dL [BEI-ACGIH] and 60 *μ*g/dL [12] recommended by the Ministry of Public Health, Thailand. However, epidemiological investigations conducted in large general population samples suggest lead may elevate blood pressure in adults at blood lead concentrations < 20 *μ*g/dL [14, 15].

### 4.2. Factors Associated with Heavy Metal Levels

A recent study found many factors influencing the increase of blood cadmium levels and blood lead levels. A result in this study shown that the mean of blood cadmium levels and blood lead levels were significantly different with different work position (such as that of garbage worker) and difference work duration [11]. Garbage workers position could be exposed with cadmium and lead more often than other positions (drivers and official workers), because of their higher contact with waste, so that their contamination with cadmium and lead would be greater than the other positions.

The result of the present study was similar with Decharat [16]. The result showed that job type was significant difference with the urinary mercury levels of garbage worker. The result was similar to the study conducted by Julander et al. [17], who reported that the median blood lead concentration of recycling workers was significantly higher than in the office workers (32 *μ*g/L; range 9.5–230 *μ*g/L) (*p* < 0.05).

In addition, a long time exposure of subject workers during worked may influence the levels of cadmium and lead in blood; accumulation of these metals can be occurring in the bodies due to a lack of practices a lack of appropriate prevention measures [18].

From what is observed in this study, PPE was used by subject workers, but type of PPE was inappropriate for this type of work. The pollutants of heavy metals can accumulate on the surface of PPE used by the subject workers such as cadmium and lead can accumulate on cotton mask, penetrate a cotton mask to inhalation pathway.

Because they were not aware of the need to clean and waited until the job was finished each day or more. With regard to PPE use, it was found that workers who used cotton mask, gloves, and aprons had significantly lower blood cadmium and blood lead levels than those who did not. Subject workers who had poor protective practices had a blood cadmium level up to 7.03 *μ*g/L (range 1.58–7.03 *μ*g/L) and a blood lead level up to 11.12 *μ*g/dL (1.98–11.12 *μ*g/L). In addition, the worker was not exposed to a blood concentration but to the cadmium concentrations in some external media. The authors noted that a subject worker regularly entered the separate area that had contacted into the body by ingestion or inhalation pathways [19].

This subject worker was exposed to up to 7.03 *μ*g/L of cadmium and 11.12 *μ*g/dL of lead for ≥8 hours per day and six days per week for 34 years. The subject worker normally did not use a cotton mask and apron and had poor personal hygiene practice. Therefore, the highest exposed worker of the group. Thus, health risk of exposure to heavy metals can be reduced by making waste technologies more contained, reducing contaminant emission, and using protective clothing [20].

Personal hygiene and behavioral risk factors were also associated with blood cadmium levels and blood lead levels. Subject workers who ate snacks during working had significantly higher blood cadmium levels and blood lead levels than those who did so sometimes. In addition, subject workers who washed their hands before the start of lunch had significantly lower blood cadmium levels and blood lead levels than those who did so sometimes. From what is observed in site area, no convenient washing facilities (with warm water and soap) were available near the collection point or work station in landfill site or for those working in the street. The result was similar to the study conducted by Rogers, 1983 [21], who suggested that the risk of exposure to heavy metals will decrease if the appropriate behaviors are adopted and practiced. Personal hygiene is also highly recommended for subject workers. German Agency for Technical Cooperation [22] reported that washing facilities were not provided for dumpsite waste pickers at the work intervals or at the end of the day. The result was similar to the study conducted by Huisman [23], who reported that the women preparing meals immediately after returning home and the women pickers bathed only once a day.

From what is observed in the areas, the authors noted that 70% of subject workers got food from the workplace and found food in the dump site. In addition, 92% water supply was used for drinking during work, and 8% carried water from their home. This result is supported by Sunthonchai and Phoolpoksin [24], who reported in terms of the demographics, characteristics, working hours, and personal hygiene behavior that most scavengers were in debt.

Oyelola et al. [25] reported that the poorest of the poor and liver on the margins of mainstream society are perceives in the scavengers in Mexico. Thus, subject workers who worked in the land fill site had encountered factors affecting health and safety. However, some factors are confirmed to be more significant and more influential than others [26–28].

In addition, the elevated lead exposure among subject workers is worrying, mainly for the women working in these settings. Children's neurodevelopment can be occurring from prenatal lead exposure, as reported by several studies [29–31].

Significant predictors of blood cadmium levels and blood lead levels included smoked cigarettes. This result is supported by Julander et al. [17], who reported that the workers who did not smoke had urinary cadmium levels significantly lower (*β* = −614, *p* = 0.001) than the smoking workers' levels. Blood cadmium levels and blood lead levels had been significantly associated with hours worked per day, days worked per week, duration of work (years), and positions. This result is supported by Julander et al. [17], who reported that the formal recycling would give rise to lower concentrations of the biomarkers. Since the workers are only exposed for eight hours per day, in plants with process ventilation. Whereas, Grant et al. [32], reported that workers are often both exposed when performing e-waste recycling and from contaminated soil, water, and locally produced food items. This study is limited by the cross-sectional study design. It is an observational study in which exposure and outcome are determined simultaneously for each subject. The sample size in this study was relatively small. Further research implementing case control approach is needed to calculate attributable risk.

## 5. Conclusion

Results in this study that the health risk behaviors of occupational life style, used PPE, and personal hygiene may endanger the health of the subject workers. Wearing of internationally recommended personal protective dressing [32] should be enforced, making the protection of eyes, respiratory airway, and skin more effective. In addition effective personnel must be maintained by provision of adequate facilities such as mild sop towel or even cleansing wipe and should be stressed in education programs.

## Figures and Tables

**Table 1:** General characteristics of the subject workers.

Parameter	Subject workers (*n* = 114)	%
Gender		
Male	85	74.6
Female	29	25.4
Age (years)		
≥20–34	66	57.9
35–44	28	24.6
45–54	20	17.5
Education level		
Secondary school	44	38.6
Vocational school	37	32.5
Diploma degree or higher	33	28.9
Income (Baht)		
<7500	76	66.7
≥7500	38	33.3
Smoked cigarettes		
Yes	64	56.1
No	50	43.9
Alcohol consumption		
Yes	66	57.9
No	48	42.1

**Table 2:** Work characteristic of the subject workers.

Parameter	Subject workers (*n* = 114)	%
Position		
Office workers	30	26.3
Drivers	30	26.3
Garbage workers	54	47.4
Duration of work (year)		
<10	17	14.9
≥10	97	85.1
Hours worked per day		
8	45	39.5
>8	69	60.5
Days worked per week		
6	82	71.9
>6	32	28.1

**Table 3:** PPEs used and personal hygiene behaviors characteristic of the subject workers.

Parameter	Subject workers (*n* = 114)	%
*PPEs used*		
Cotton mask		
No	29	25.4
Yes	85	74.6
Gloves		
No	73	64.0
Yes	41	36.0
Aprons		
No	45	39.5
Yes	69	60.5
*Personal hygiene of workers*		
Ate snacks or drank water at work area		
Sometimes	60	52.6
Always	54	47.4
Washed hands before lunch		
Sometimes	47	41.2
Always	67	58.8
Changed clothes after work		
Sometimes	72	63.2
Always	42	36.8

**Table 4:** Comparison of blood cadmium levels and blood lead levels in subject workers.

Parameter	Mean ± SD of subjects workers (*n* = 114)	Range
Blood cadmium levels (*µ*g/L)	2.95 ± 0.58	1.58–7.03
Blood lead levels (*µ*g/dL)	8.58 ± 2.58	1.98–11.12

**Table 5:** Variables related to subject workers' blood cadmium levels and blood lead levels.

Parameter	Subject workers (*n* = 114)	Blood cadmium levels (*µ*g/L) Mean ± SD	95% CI	*p* value	Blood lead levels (*µ*g/dL) Mean ± SD	95% CI	*p* value
Gender							
Male	85 (74.6)	1.96 ± 1.20	1.59–2.11	<0.001^*∗*^	8.57 ± 1.48	2.25–2.89	<0.001^*∗*^
Female	29 (25.4)	1.39 ± 0.74	1.30–1.87		3.21 ± 0.46	1.65–2.77	
Age (years)							
≥20–34	66 (57.9)	1.82 ± 1.13	1.70–1.50	0.258	3.37 ± 1.24	1.97–2.58	0.524
35–44	28 (24.6)	1.65 ± 0.98	1.27–2.04		3.70 ± 1.72	1.93–3.27	
45–54	20 (17.5)	1.85 ± 1.20	1.28–2.41		4.00 ± 1.80	2.15–3.84	
Education level							
Secondary school	33 (28.9)	1.53 ± 1.06	1.15–1.90	0.412	4.79 ± 1.78	2.15–3.42	0.127
Vocational school	37 (32.5)	1.83 ± 1.08	1.46–2.19		3.96 ± 2.03	2.05–3.08	
Diploma degree or higher	44 (38.6)	1.93 ± 1.14	1.58–2.29		4.38 ± 1.14	1.83–3.53	
Income (Baht)							
<7500	76 (66.7)	1.54 ± 0.15	1.64–2.17	<0.001^*∗*^	2.63 ± 1.49	2.21–2.89	<0.001^*∗*^
≥7500	38 (33.3)	2.00 ± 0.98	1.21–1.86		7.55 ± 1.49	1.85–4.83	
Smoked cigarettes							
Yes	50 (43.9)	2.79 ± 0.93	1.52–2.05	<0.001^*∗*^	8.94 ± 1.58	2.49–4.38	<0.001
No	64 (56.1)	1.78 ± 1.23	1.47–2.09		3.12 ± 1.32	1.79–2.45	
Alcohol consumption							
Yes	66 (57.9)	1.73 ± 1.00	1.51–2.23	0.492	4.61 ± 1.45	2.24–2.96	0.309
No	48 (42.1)	1.87 ± 1.24			4.32 ± 1.53	1.86–2.76	
Position							
Office workers	30 (26.3)	1.40 ± 0.71	1.22–1.58	<0.001^*∗*^	4.45 ± 1.56	2.04–2.85	0.001^*∗*^
Drivers	30 (26.3)	2.21 ± 1.30	1.86–2.57		8.52 ± 1.41	2.13–4.90	
Garbage workers	54 (47.4)	4.05 ± 1.54	2.10–0.58		10.58 ± 2.41	1.90–4.28	
Duration of work (year)							
<10	17 (14.9)	1.15 ± 0.58	0.85–1.46	<0.001^*∗*^	3.05 ± 1.29	1.39–2.72	<0.001^*∗*^
≥10	97 (85.1)	1.89 ± 1.14	1.67–2.12		8.55 ± 2.30	2.25–3.86	
Hours worked per day							
8	45 (39.5)	1.68 ± 1.05	1.37–2.00	0.438	3.20 ± 1.40	1.78–2.63	<0.001^*∗*^
>8	69 (60.5)	1.85 ± 1.14	1.57–2.12		7.67 ± 1.52	2.30–3.03	
Days worked per week							
6	82 (71.9)	1.70 ± 1.10	1.45–1.94	0.188	4.28 ± 1.34	1.98–4.57	0.001^*∗*^
>6	32 (28.1)	2.00 ± 1.09	1.61–2.40		8.01 ± 1.73	2.38–3.63	
*PPEs used*							
Cotton mask							
No	29 (25.4)	1.81 ± 1.11	1.57–2.01	<0.001^*∗*^	8.31 ± 1.36	2.01–4.59	<0.001^*∗*^
Yes	85 (74.6)	0.71 ± 1.10	1.29–2.13		2.33 ± 1.76	2.33–3.67	
Gloves							
No	73 (64.0)	1.83 ± 1.06	1.33–2.09	<0.001^*∗*^	8.28 ± 1.52	1.79–4.75	<0.001^*∗*^
Yes	41 (36.0)	1.71 ± 1.18	1.58–2.08		2.60 ± 1.46	2.25–3.94	
Aprons							
No	45 (39.5)	1.77 ± 1.13	1.44–1.99	0.258	4.55 ± 1.50	2.10–4.78	0.478
Yes	69 (60.5)	1.89 ± 1.06	1.57–2.21		4.43 ± 1.49	1.07–2.79	
*Personal hygiene of workers*							
Ate snacks or drank water at work area							
Sometimes	60 (52.6)	2.08 ± 1.12	1.78–2.38	<0.001^*∗*^	7.65 ± 1.48	2.26–3.03	<0.001^*∗*^
Always	54 (47.4)	1.06 ± 0.99	1.18–1.73		2.30 ± 1.49	1.89–2.70	
Washed hands before lunch							
Sometimes	47 (41.2)	2.02 ± 1.23	1.65–2.39	<0.001^*∗*^	7.49 ± 1.50	2.05–4.93	<0.001^*∗*^
Always	67 (58.8)	1.02 ± 0.98	1.38–1.87		4.49 ± 1.49	2.11–3.84	
Changed clothes after work							
Sometimes	42 (36.8)	1.98 ± 1.17	1.61–2.35	<0.001^*∗*^	8.54 ± 1.48	2.08–4.91	<0.001^*∗*^
Always	72 (63.2)	1.07 ± 1.05	1.42–1.92		4.44 ± 1.50	2.09–3.80	

^*∗*^Significant at *p* < 0.05.

**Table 6:** Multiple regression occupational life style, used PPE, and personal hygiene behavior on blood cadmium and lead levels in subject workers (*n* = 114).

Dependent variable, independent variable	Blood cadmium levels in subject workers				Blood lead levels in subject workers			
	Adjusted *R* ^2^	Standardized beta coefficient	*t* value	Significance	Adjusted *R* ^2^	Standardized beta coefficient	*t* value	Significance
*Equation *1								
Smoking	0.418	0.341	4.213	0.001^*∗*^	0.450	0.452	4.402	0.001^*∗*^
Days worked per week		0.432	4.521	0.001^*∗*^		0.021	0.112	0.743
Duration of work		0.039	0.401	0.521		0.055	0.492	0.674
Position		0.448	5.120	0.001^*∗*^		0.466	4.480	0.001^*∗*^
Mask		−0.921	−8.927	0.001^*∗*^		−0.721	−6.989	0.001^*∗*^
*Equation *2								
Smoking	0.458	−0.018	−0.112	0.897	0.378	0.032	0.622	0.574
Days worked per week		0.511	5.200	0.001^*∗*^		0.062	0.395	0.544
Duration of work		0.095	0.897	0.450		0.118	1.850	0.095
Position		0.158	2.058	0.312		0.395	3.801	0.001^*∗*^
Gloves		−0.358	−2.991	0.031^*∗*^		−0.488	−4.291	0.011^*∗*^
*Equation *3								
Smoking	0.401	0.445	4.810	0.001^*∗*^	0.530	−0.068	−0.784	0.501
Days worked per week		0.025	0.251	0.410		0.408	4.585	0.001^*∗*^
Duration of work		0.075	0.870	0.390		0.382	3.854	0.001^*∗*^
Position		0.091	0.955	0.420		0.145	1.452	0.148
Ate snacks or drank water at work		−0.658	−7.982	0.001^*∗*^		0.565	−2.521	0.021^*∗*^
*Equation *4								
Smoking	0.569	0.501	5.220	0.001^*∗*^	0.391	−0.085	−1.009	0.251
Days worked per week		0.691	6.852	0.001^*∗*^		0.410	4.210	0.001^*∗*^
Duration of work		0.051	0.511	0.721		0.382	3.791	0.001^*∗*^
Position		0.495	5.233	0.001^*∗*^		0.325	3.180	0.001^*∗*^
Washed hands before lunch		−0.600	−7.882	0.001^*∗*^		0.004	0.072	0.985

^*∗*^Significance at *p* < 0.05.

## References

[1] Johannessen L. M., Boyer G. (1999). *Observations of Solid Waste Landfills in Developing Countries: Africa, Asia, and Latin America*.

[2] Daniel H., Perinaz B. T. (2012). *What a Waste: A Global Review of Solid Waste Management*.

[3] Bangkok Metropolitan Administration (2012). *Action Plan on Global Warming Mitigation 2007–2012*.

[4] Cointreau S. Occupational and Environmental Health Issues of Solid Waste Management Special Emphasis on Middle- and Lower-Income Countries. http://siteresources.worldbank.org/INTUSWM/Resources/up-2.pdf.

[5] Ray M. R., Mukherjee G., Roychoudhury S., Lahiri T., Roy S. (2005). Respiratory and general health impairments of workers employed in a municipal solid waste disposal at an open landfill site in Delhi. *International Journal of Hygiene and Environmental Health*.

[6] Poulsen O. M., Breum N. O., Ebbehøj N. (1995). Collection of domestic waste: review of occupational health problems and their possible causes. *Science of the Total Environment*.

[7] Poulsen O. M., Midtgard U. Bioaerosol exposure and health problems.

[8] Malkin R., Brandt-Rauf P., Graziano J., Parides M. (1992). Blood lead levels in incinerator workers. *Environmental Research*.

[9] Ramesh A., Koziński J. A. (2001). Investigations of ash topography/morphology and their relationship with heavy metals leachability. *Environmental Pollution*.

[10] National Institute for Occupational Safety and Health (NIOSH) (1994). Method 8003: lead in blood and urine. *NIOSH Manual of Analytical Methods (NMAM)*.

[11] Nordberg G. F., Fowler B. A., Nordberg M., Friberg L. T. (2007). *Handbook on the Toxicology of Metals*.

[12] American Conference of Governmental Industrial Hygienists (2011). *TLVs and BEIs: Threshold Limit Values for Chemical Substances and Physical Agents & Biological Exposure Indices*.

[13] Alfvén T., Järup L., Elinder C.-G. (2002). Cadmium and lead in blood in relation to low bone mineral density and tubular proteinuria. *Environmental Health Perspectives*.

[14] Nash D., Magder L., Lustberg M. (2003). Blood lead, blood pressure, and hypertension in perimenopausal and postmenopausal women. *The Journal of the American Medical Association*.

[15] Schwartz J. (1988). The relationship between blood lead and blood pressure in the NHANES II survey. *Environmental Health Perspectives*.

[16] Decharat S. (2012). Mercury exposure among garbage workers in Southern Thailand. *Safety and Health at Work*.

[17] Julander A., Lundgren L., Skare L. (2014). Formal recycling of e-waste leads to increased exposure to toxic metals: an occupational exposure study from Sweden. *Environment International*.

[18] Järup L. (2003). Hazards of heavy metal contamination. *British Medical Bulletin*.

[19] Johri N., Jacquillet G., Unwin R. (2010). Heavy metal poisoning: the effects of cadmium on the kidney. *BioMetals*.

[20] Varma R. A., Babu A., Vinod T. R., Ravindran K. V., Sabu T. Status of municipal solid waste generation and technology options for treatment with special reference to Kerala.

[21] Rogers R. W., Cacioppo J. T., Petty R. (1983). Cognitive and physiological processes in fear appeals and attitude change: a revised theory of protection motivation. *Social Psychophysiology*.

[22] German Agency for Technical Cooperation (1986). *Scavenger Activities and Health Hazards to Scavengers*.

[23] Huisman M., Baud I., Schenk H. (1994). The position of waste pickers in solid waste management. *Solid Waste Management*.

[24] Sunthonchai S., Phoolpoksin W. Health and environment protection of waste picker and related labors. *Complete Report*.

[25] Oyelola O. T., Babatunde A. I., Odunlade A. K. (2008). Health implications of solid waste disposal: case study of Olusosun dumpsite, Lagos, Nigeria. *International Journal of Pure and Applied Sciences*.

[26] Khalil A., Milhem M. (2004). *Investigation of Occupational Health and Safety Hazards among Domestic Waste Collectors in Bethlehem and Hebron Districts*.

[27] Ray M. R., Mukherjee G., Roychowdhury S., Lahiri T. (2004). Respiratory and general health impairments of ragpickers in India: a study in Delhi. *International Archives of Occupational and Environmental Health*.

[28] Furedy C. Women and solid wastes in poor communities.

[29] Bellinger D. C. (2013). Prenatal exposures to environmental chemicals and children's neurodevelopment: an update. *Safety and Health at Work*.

[30] Bellinger D., Leviton A., Waternaux C., Needleman H., Rabinowitz M. (1987). Longitudinal analyses of prenatal and postnatal lead exposure and early cognitive development. *The New England Journal of Medicine*.

[31] Grandjean P., Landrigan P. (2006). Developmental neurotoxicity of industrial chemicals. *The Lancet*.

[32] Grant K., Goldizen F. C., Sly P. D. (2013). Health consequences of exposure to e-waste: a systematic review. *The Lancet Global Health*.

